# Supplementary material for: A pair of non-optimal codons are necessary for the correct biosynthesis of the Aspergillus nidulans urea transporter, UreA
Source: R Soc Open Sci. 2019 Nov 13;6(11):190773. doi: 10.1098/rsos.190773 (PMC6894576; doi:10.1098/rsos.190773)
Supplement: Supplementary material Sanguinetti et al. [file rsos190773supp1.pdf]

SUPPLEMENTARY MATERIAL

A pair of nonoptimal codons are necessary for the correct biosynthesis of the *Aspergillus nidulans* urea transporter, UreA

Manuel Sanguinetti, Andrés Iriarte, Sotiris Amillis, Mónica Marín, Héctor Musto and Ana Ramón*

* Corresponding author; e-mail: anaramon@fcien.edu.uy

Aspergillus nidulans

```
# ni.387 Length: 693
# ni.387 Number of predicted TMHs:  15
# ni.387 Exp number of AAs in TMHs: 331.55861
# ni.387 Exp number, first 60 AAs:  22.78375
# ni.387 Total prob of N-in:        0.01305
# ni.387 POSSIBLE N-term signal sequence
ni.387  TMHMM2.0       outside      1    24
ni.387  TMHMM2.0       TMhelix     25    47
ni.387  TMHMM2.0       inside      48    67
ni.387  TMHMM2.0       TMhelix     68    87
ni.387  TMHMM2.0       outside     88   101
ni.387  TMHMM2.0       TMhelix    102   124
ni.387  TMHMM2.0       inside     125   144
ni.387  TMHMM2.0       TMhelix    145   167
ni.387  TMHMM2.0       outside    168   176
ni.387  TMHMM2.0       TMhelix    177   199
ni.387  TMHMM2.0       inside     200   211
ni.387  TMHMM2.0       TMhelix    212   229
ni.387  TMHMM2.0       outside    230   267
ni.387  TMHMM2.0       TMhelix    268   290
ni.387  TMHMM2.0       inside     291   301
ni.387  TMHMM2.0       TMhelix    302   324
ni.387  TMHMM2.0       outside    325   363
ni.387  TMHMM2.0       TMhelix    364   386
ni.387  TMHMM2.0       inside     387   406
ni.387  TMHMM2.0       TMhelix    407   429
ni.387  TMHMM2.0       outside    430   433
ni.387  TMHMM2.0       TMhelix    434   456
ni.387  TMHMM2.0       inside     457   460
ni.387  TMHMM2.0       TMhelix    461   483
ni.387  TMHMM2.0       outside    484   502
ni.387  TMHMM2.0       TMhelix    503   525
ni.387  TMHMM2.0       inside     526   583
ni.387  TMHMM2.0       TMhelix    584   606
ni.387  TMHMM2.0       outside    607   615
ni.387  TMHMM2.0       TMhelix    616   638
ni.387  TMHMM2.0       inside     639   693
```

Aspergillus oryzae

```
# or.1414 Length: 693
# or.1414 Number of predicted TMHs:  15
# or.1414 Exp number of AAs in TMHs: 335.13636
# or.1414 Exp number, first 60 AAs:  25.62461
# or.1414 Total prob of N-in:        0.00078
# or.1414 POSSIBLE N-term signal sequence
or.1414 TMHMM2.0       outside      1    14
or.1414 TMHMM2.0       TMhelix     15    37
or.1414 TMHMM2.0       inside      38    56
or.1414 TMHMM2.0       TMhelix     57    76
or.1414 TMHMM2.0       outside     77    90
or.1414 TMHMM2.0       TMhelix     91   113
or.1414 TMHMM2.0       inside     114   133
or.1414 TMHMM2.0       TMhelix    134   156
or.1414 TMHMM2.0       outside    157   165
or.1414 TMHMM2.0       TMhelix    166   188
or.1414 TMHMM2.0       inside     189   200
or.1414 TMHMM2.0       TMhelix    201   218
or.1414 TMHMM2.0       outside    219   256
or.1414 TMHMM2.0       TMhelix    257   279
or.1414 TMHMM2.0       inside     280   299
or.1414 TMHMM2.0       TMhelix    300   322
or.1414 TMHMM2.0       outside    323   370
or.1414 TMHMM2.0       TMhelix    371   393
or.1414 TMHMM2.0       inside     394   412
or.1414 TMHMM2.0       TMhelix    413   435
or.1414 TMHMM2.0       outside    436   439
or.1414 TMHMM2.0       TMhelix    440   462
or.1414 TMHMM2.0       inside     463   468
or.1414 TMHMM2.0       TMhelix    469   491
or.1414 TMHMM2.0       outside    492   510
or.1414 TMHMM2.0       TMhelix    511   533
or.1414 TMHMM2.0       inside     534   587
or.1414 TMHMM2.0       TMhelix    588   610
or.1414 TMHMM2.0       outside    611   624
or.1414 TMHMM2.0       TMhelix    625   647
or.1414 TMHMM2.0       inside     648   693
```

Aspergillus terreus

```
# te.2629 Length: 679
# te.2629 Number of predicted TMHs:  15
# te.2629 Exp number of AAs in TMHs: 335.03307
# te.2629 Exp number, first 60 AAs:  25.59071
# te.2629 Total prob of N-in:        0.00189
# te.2629 POSSIBLE N-term signal sequence
te.2629 TMHMM2.0       outside      1    14
te.2629 TMHMM2.0       TMhelix     15    37
te.2629 TMHMM2.0       inside      38    56
te.2629 TMHMM2.0       TMhelix     57    76
te.2629 TMHMM2.0       outside     77    90
te.2629 TMHMM2.0       TMhelix     91   113
te.2629 TMHMM2.0       inside     114   133
te.2629 TMHMM2.0       TMhelix    134   156
te.2629 TMHMM2.0       outside    157   165
te.2629 TMHMM2.0       TMhelix    166   188
te.2629 TMHMM2.0       inside     189   200
te.2629 TMHMM2.0       TMhelix    201   223
te.2629 TMHMM2.0       outside    224   257
te.2629 TMHMM2.0       TMhelix    258   280
te.2629 TMHMM2.0       inside     281   292
te.2629 TMHMM2.0       TMhelix    293   315
te.2629 TMHMM2.0       outside    316   352
te.2629 TMHMM2.0       TMhelix    353   375
te.2629 TMHMM2.0       inside     376   395
te.2629 TMHMM2.0       TMhelix    396   418
te.2629 TMHMM2.0       outside    419   422
te.2629 TMHMM2.0       TMhelix    423   445
te.2629 TMHMM2.0       inside     446   451
te.2629 TMHMM2.0       TMhelix    452   471
te.2629 TMHMM2.0       outside    472   490
te.2629 TMHMM2.0       TMhelix    491   513
te.2629 TMHMM2.0       inside     514   568
te.2629 TMHMM2.0       TMhelix    569   591
te.2629 TMHMM2.0       outside    592   605
te.2629 TMHMM2.0       TMhelix    606   628
te.2629 TMHMM2.0       inside     629   679
```

Neosartorya fischeri

```
# nf.1793 Length: 679
# nf.1793 Number of predicted TMHs:  15
# nf.1793 Exp number of AAs in TMHs: 333.6653
# nf.1793 Exp number, first 60 AAs:  24.95619
# nf.1793 Total prob of N-in:        0.00082
# nf.1793 POSSIBLE N-term signal sequence
nf.1793 TMHMM2.0       outside      1    14
nf.1793 TMHMM2.0       TMhelix     15    37
nf.1793 TMHMM2.0       inside      38    57
nf.1793 TMHMM2.0       TMhelix     58    77
nf.1793 TMHMM2.0       outside     78    91
nf.1793 TMHMM2.0       TMhelix     92   114
nf.1793 TMHMM2.0       inside     115   134
nf.1793 TMHMM2.0       TMhelix    135   157
nf.1793 TMHMM2.0       outside    158   166
nf.1793 TMHMM2.0       TMhelix    167   189
nf.1793 TMHMM2.0       inside     190   201
nf.1793 TMHMM2.0       TMhelix    202   221
nf.1793 TMHMM2.0       outside    222   257
nf.1793 TMHMM2.0       TMhelix    258   280
nf.1793 TMHMM2.0       inside     281   292
nf.1793 TMHMM2.0       TMhelix    293   315
nf.1793 TMHMM2.0       outside    316   353
nf.1793 TMHMM2.0       TMhelix    354   376
nf.1793 TMHMM2.0       inside     377   395
nf.1793 TMHMM2.0       TMhelix    396   418
nf.1793 TMHMM2.0       outside    419   422
nf.1793 TMHMM2.0       TMhelix    423   445
nf.1793 TMHMM2.0       inside     446   451
nf.1793 TMHMM2.0       TMhelix    452   474
nf.1793 TMHMM2.0       outside    475   493
nf.1793 TMHMM2.0       TMhelix    494   516
nf.1793 TMHMM2.0       inside     517   569
nf.1793 TMHMM2.0       TMhelix    570   592
nf.1793 TMHMM2.0       outside    593   601
nf.1793 TMHMM2.0       TMhelix    602   624
nf.1793 TMHMM2.0       inside     625   679
```

Aspergillus clavatus

```
# cl.2744 Length: 679
# cl.2744 Number of predicted TMHs:  15
# cl.2744 Exp number of AAs in TMHs: 332.73698
# cl.2744 Exp number, first 60 AAs:  24.9873
# cl.2744 Total prob of N-in:        0.00105
# cl.2744 POSSIBLE N-term signal sequence
cl.2744 TMHMM2.0       outside      1    14
cl.2744 TMHMM2.0       TMhelix     15    37
cl.2744 TMHMM2.0       inside      38    57
cl.2744 TMHMM2.0       TMhelix     58    77
cl.2744 TMHMM2.0       outside     78    91
cl.2744 TMHMM2.0       TMhelix     92   114
cl.2744 TMHMM2.0       inside     115   134
cl.2744 TMHMM2.0       TMhelix    135   157
cl.2744 TMHMM2.0       outside    158   166
cl.2744 TMHMM2.0       TMhelix    167   189
cl.2744 TMHMM2.0       inside     190   201
cl.2744 TMHMM2.0       TMhelix    202   219
cl.2744 TMHMM2.0       outside    220   257
cl.2744 TMHMM2.0       TMhelix    258   280
cl.2744 TMHMM2.0       inside     281   292
cl.2744 TMHMM2.0       TMhelix    293   315
cl.2744 TMHMM2.0       outside    316   353
cl.2744 TMHMM2.0       TMhelix    354   376
cl.2744 TMHMM2.0       inside     377   395
cl.2744 TMHMM2.0       TMhelix    396   418
cl.2744 TMHMM2.0       outside    419   422
cl.2744 TMHMM2.0       TMhelix    423   445
cl.2744 TMHMM2.0       inside     446   451
cl.2744 TMHMM2.0       TMhelix    452   474
cl.2744 TMHMM2.0       outside    475   493
cl.2744 TMHMM2.0       TMhelix    494   516
cl.2744 TMHMM2.0       inside     517   568
cl.2744 TMHMM2.0       TMhelix    569   591
cl.2744 TMHMM2.0       outside    592   605
cl.2744 TMHMM2.0       TMhelix    606   628
cl.2744 TMHMM2.0       inside     629   679
```

Aspergillus niger

```
# ng.4333 Length: 668
# ng.4333 Number of predicted TMHs:  15
# ng.4333 Exp number of AAs in TMHs: 331.47825
# ng.4333 Exp number, first 60 AAs:  25.28513
# ng.4333 Total prob of N-in:        0.06193
# ng.4333 POSSIBLE N-term signal sequence
ng.4333 TMHMM2.0       outside      1    14
ng.4333 TMHMM2.0       TMhelix     15    37
ng.4333 TMHMM2.0       inside      38    60
ng.4333 TMHMM2.0       TMhelix     61    78
ng.4333 TMHMM2.0       outside     79    82
ng.4333 TMHMM2.0       TMhelix     83   102
ng.4333 TMHMM2.0       inside     103   121
ng.4333 TMHMM2.0       TMhelix    122   144
ng.4333 TMHMM2.0       outside    145   153
ng.4333 TMHMM2.0       TMhelix    154   176
ng.4333 TMHMM2.0       inside     177   182
ng.4333 TMHMM2.0       TMhelix    183   205
ng.4333 TMHMM2.0       outside    206   245
ng.4333 TMHMM2.0       TMhelix    246   268
ng.4333 TMHMM2.0       inside     269   279
ng.4333 TMHMM2.0       TMhelix    280   302
ng.4333 TMHMM2.0       outside    303   341
ng.4333 TMHMM2.0       TMhelix    342   364
ng.4333 TMHMM2.0       inside     365   383
ng.4333 TMHMM2.0       TMhelix    384   406
ng.4333 TMHMM2.0       outside    407   410
ng.4333 TMHMM2.0       TMhelix    411   433
ng.4333 TMHMM2.0       inside     434   439
ng.4333 TMHMM2.0       TMhelix    440   462
ng.4333 TMHMM2.0       outside    463   481
ng.4333 TMHMM2.0       TMhelix    482   504
ng.4333 TMHMM2.0       inside     505   556
ng.4333 TMHMM2.0       TMhelix    557   579
ng.4333 TMHMM2.0       outside    580   593
ng.4333 TMHMM2.0       TMhelix    594   616
ng.4333 TMHMM2.0       inside     617   668
```

Aspergillus fumigatus

```
# fu.474 Length: 664
# fu.474 Number of predicted TMHs:  15
# fu.474 Exp number of AAs in TMHs: 332.7963
# fu.474 Exp number, first 60 AAs:  24.95602
# fu.474 Total prob of N-in:        0.00083
# fu.474 POSSIBLE N-term signal sequence
fu.474  TMHMM2.0       outside      1    14
fu.474  TMHMM2.0       TMhelix     15    37
fu.474  TMHMM2.0       inside      38    57
fu.474  TMHMM2.0       TMhelix     58    77
fu.474  TMHMM2.0       outside     78    91
fu.474  TMHMM2.0       TMhelix     92   114
fu.474  TMHMM2.0       inside     115   134
fu.474  TMHMM2.0       TMhelix    135   157
fu.474  TMHMM2.0       outside    158   166
fu.474  TMHMM2.0       TMhelix    167   189
fu.474  TMHMM2.0       inside     190   201
fu.474  TMHMM2.0       TMhelix    202   221
fu.474  TMHMM2.0       outside    222   259
fu.474  TMHMM2.0       TMhelix    260   282
fu.474  TMHMM2.0       inside     283   286
fu.474  TMHMM2.0       TMhelix    287   309
fu.474  TMHMM2.0       outside    310   323
fu.474  TMHMM2.0       TMhelix    324   346
fu.474  TMHMM2.0       inside     347   378
fu.474  TMHMM2.0       TMhelix    379   398
fu.474  TMHMM2.0       outside    399   401
fu.474  TMHMM2.0       TMhelix    402   424
fu.474  TMHMM2.0       inside     425   436
fu.474  TMHMM2.0       TMhelix    437   459
fu.474  TMHMM2.0       outside    460   478
fu.474  TMHMM2.0       TMhelix    479   501
fu.474  TMHMM2.0       inside     502   553
fu.474  TMHMM2.0       TMhelix    554   576
fu.474  TMHMM2.0       outside    577   590
fu.474  TMHMM2.0       TMhelix    591   613
fu.474  TMHMM2.0       inside     614   664
```

Aspergillus flavus

```
# fl.2167 Length: 683
# fl.2167 Number of predicted TMHs:  13
# fl.2167 Exp number of AAs in TMHs: 319.4979
# fl.2167 Exp number, first 60 AAs:  23.21413
# fl.2167 Total prob of N-in:        0.26231
# fl.2167 POSSIBLE N-term signal sequence
fl.2167 TMHMM2.0       outside      1    14
fl.2167 TMHMM2.0       TMhelix     15    37
fl.2167 TMHMM2.0       inside      38   118
fl.2167 TMHMM2.0       TMhelix    119   141
fl.2167 TMHMM2.0       outside    142   150
fl.2167 TMHMM2.0       TMhelix    151   173
fl.2167 TMHMM2.0       inside     174   179
fl.2167 TMHMM2.0       TMhelix    180   202
fl.2167 TMHMM2.0       outside    203   242
fl.2167 TMHMM2.0       TMhelix    243   265
fl.2167 TMHMM2.0       inside     266   285
fl.2167 TMHMM2.0       TMhelix    286   308
fl.2167 TMHMM2.0       outside    309   356
fl.2167 TMHMM2.0       TMhelix    357   379
fl.2167 TMHMM2.0       inside     380   398
fl.2167 TMHMM2.0       TMhelix    399   421
fl.2167 TMHMM2.0       outside    422   425
fl.2167 TMHMM2.0       TMhelix    426   448
fl.2167 TMHMM2.0       inside     449   454
fl.2167 TMHMM2.0       TMhelix    455   477
fl.2167 TMHMM2.0       outside    478   498
fl.2167 TMHMM2.0       TMhelix    499   521
fl.2167 TMHMM2.0       inside     522   576
fl.2167 TMHMM2.0       TMhelix    577   599
fl.2167 TMHMM2.0       outside    600   613
fl.2167 TMHMM2.0       TMhelix    614   636
fl.2167 TMHMM2.0       inside     637   683
```

Supplementary data S1. *TMHMM prediction of transmembrane helixes in A. nidulans UreA and its orthologues in A. oryzae, A. terreus, N. fischeri, A. clavatus, A. niger, A. fumigatus and A. flavus*

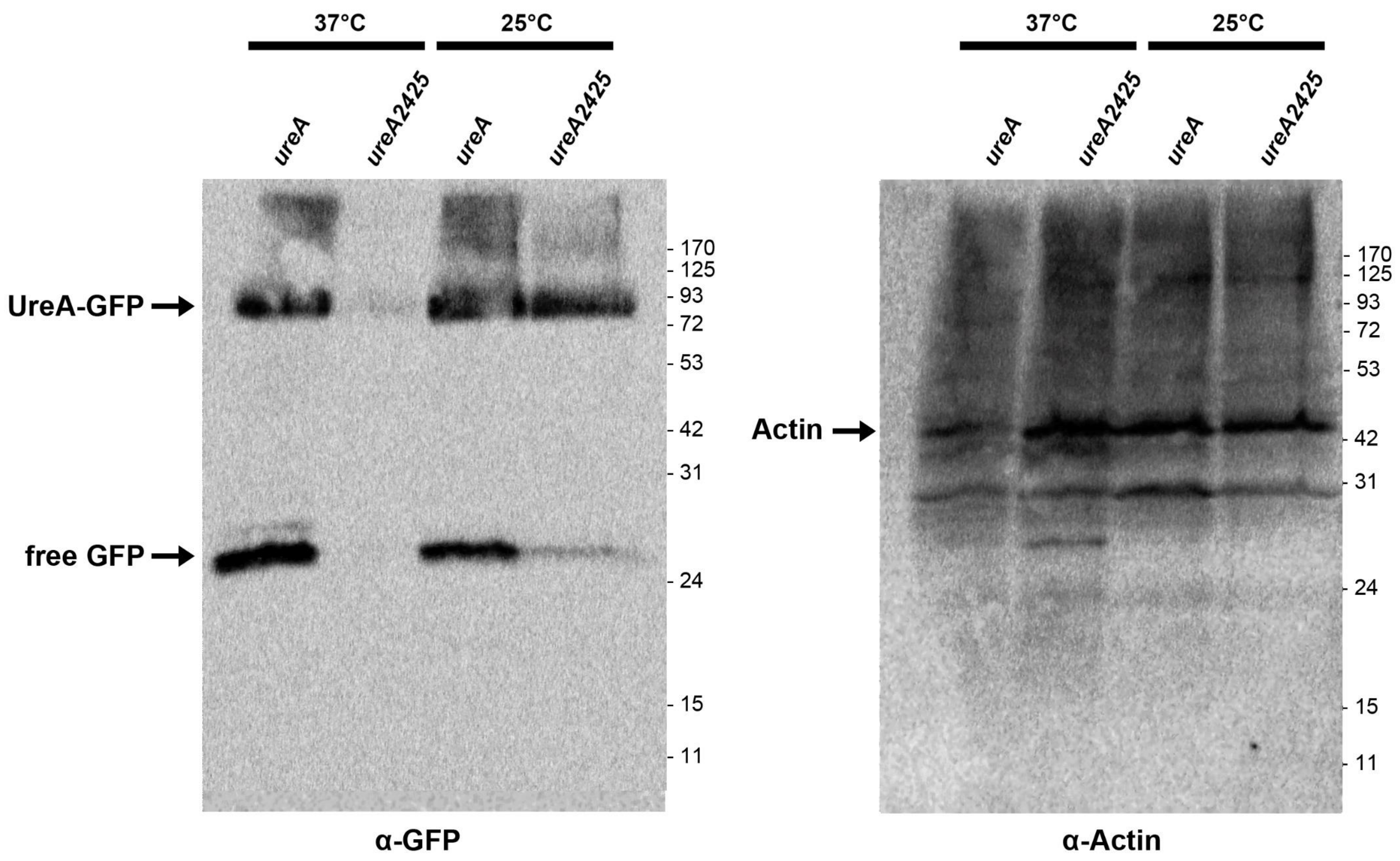

Supplementary Figure S1. Full length Western blots corresponding to Figure 4b. Blots were done on total protein extracts of UreA-GFP wild type and synonymous mutants from cultures grown at 37ºC or 25ºC, and incubated with anti-GFP antibody (left) and anti-actin antibody (right). See main text for details. Molecular marker bands are in KDa.

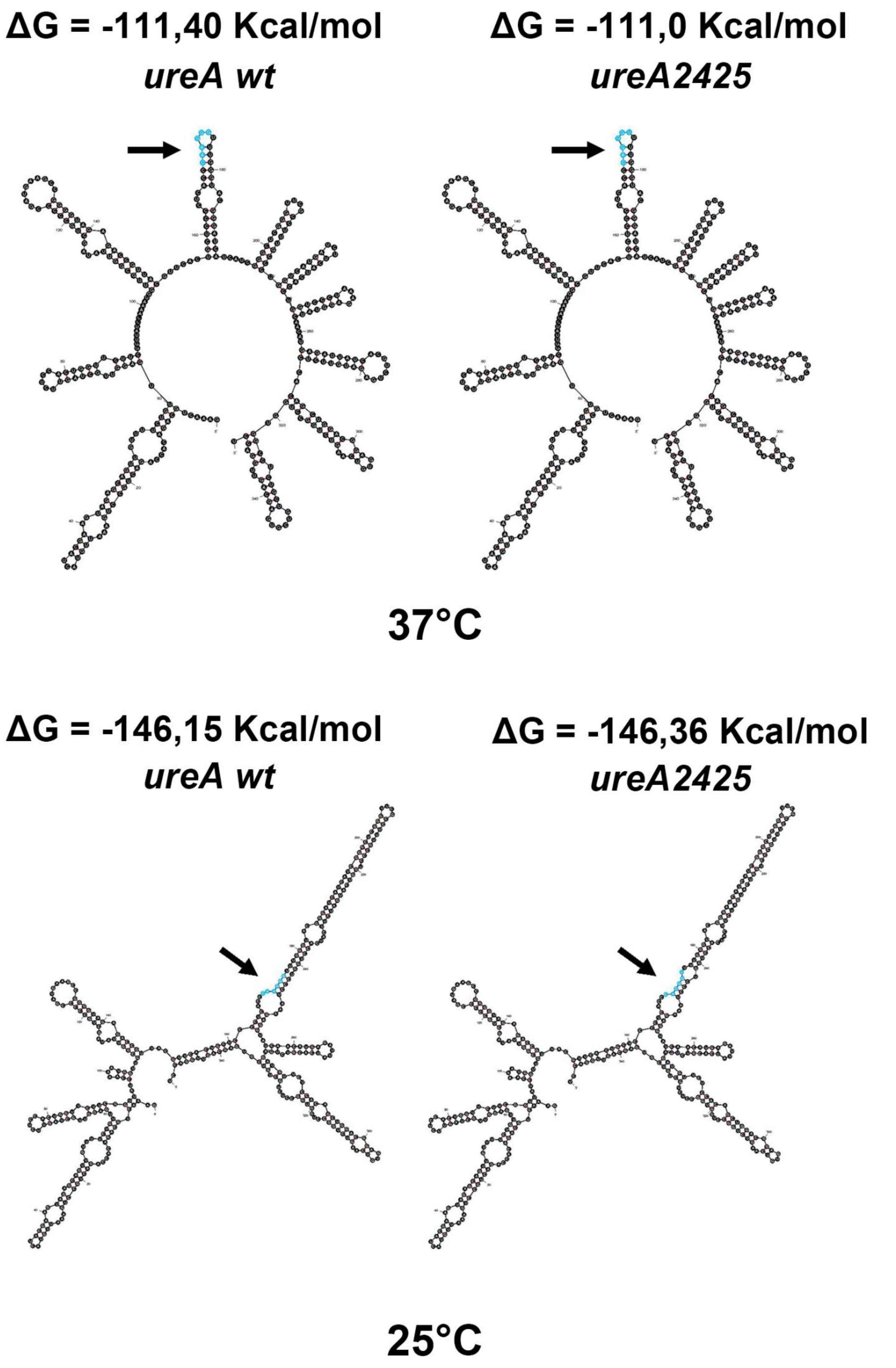

Supplementary Figure S2. Secondary structure prediction of *ureA2425* mRNA. Secondary structure predictions of *wt ureA* and *ureA2425* mRNA using mfold online software (see M&M). As input a sequence including 100 nucleotide upstream of the ATG start codon and 250 nucleotides downstream was used. Prediction was performed at 25 and 37°C. Minimum free energies (ΔGs) are indicated. Arrows indicate de position of codons 24 and 25 within the structures shown.

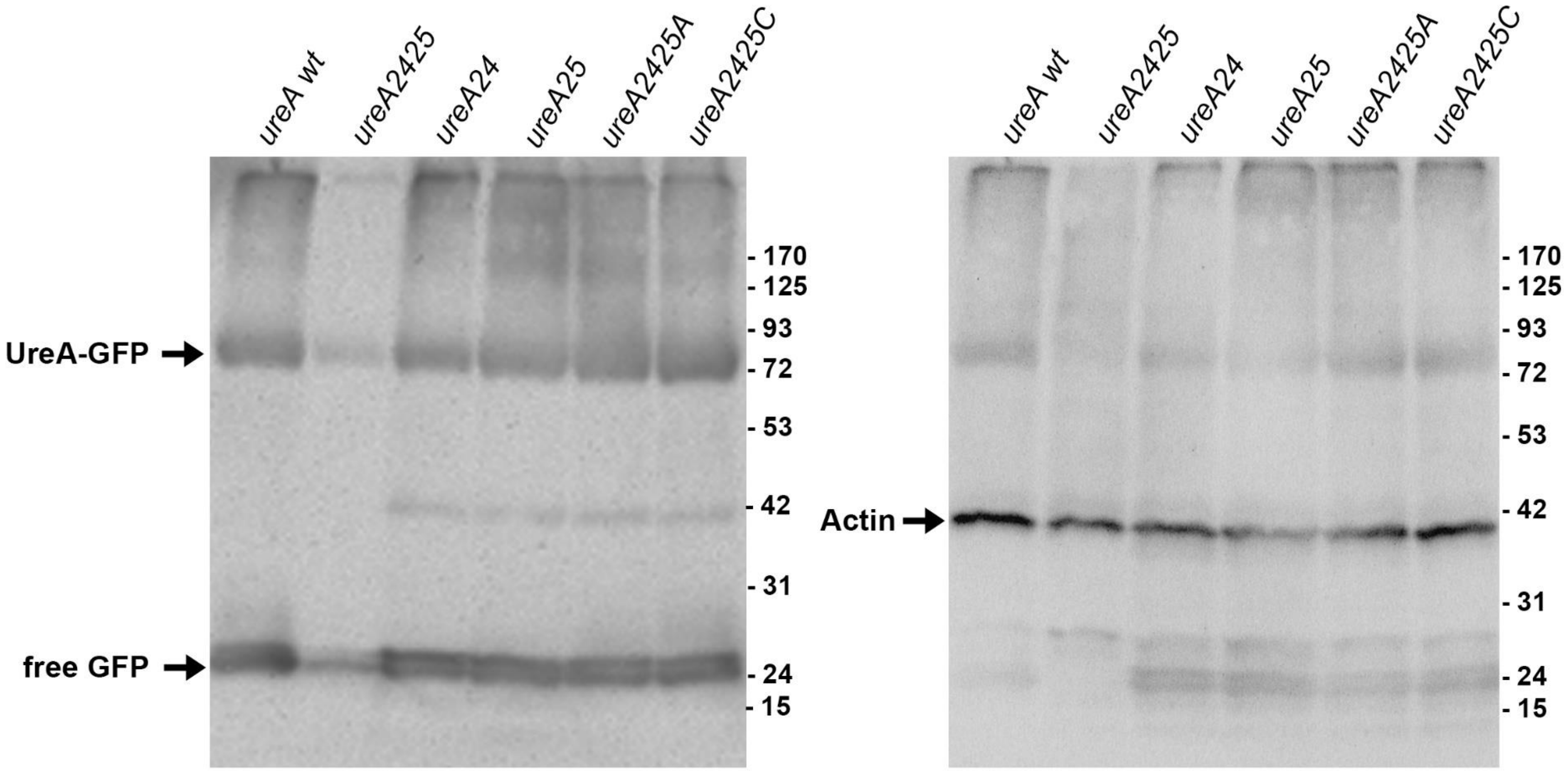

Supplementary Figure S3. Western blot analysis on total protein extracts of UreA-GFP wild type and synonymous mutants incubated with anti-GFP antibody (left) and anti-actin antibody (right). Full-length blots are presented. See main text for details. Molecular weight marker bands are in KDa.

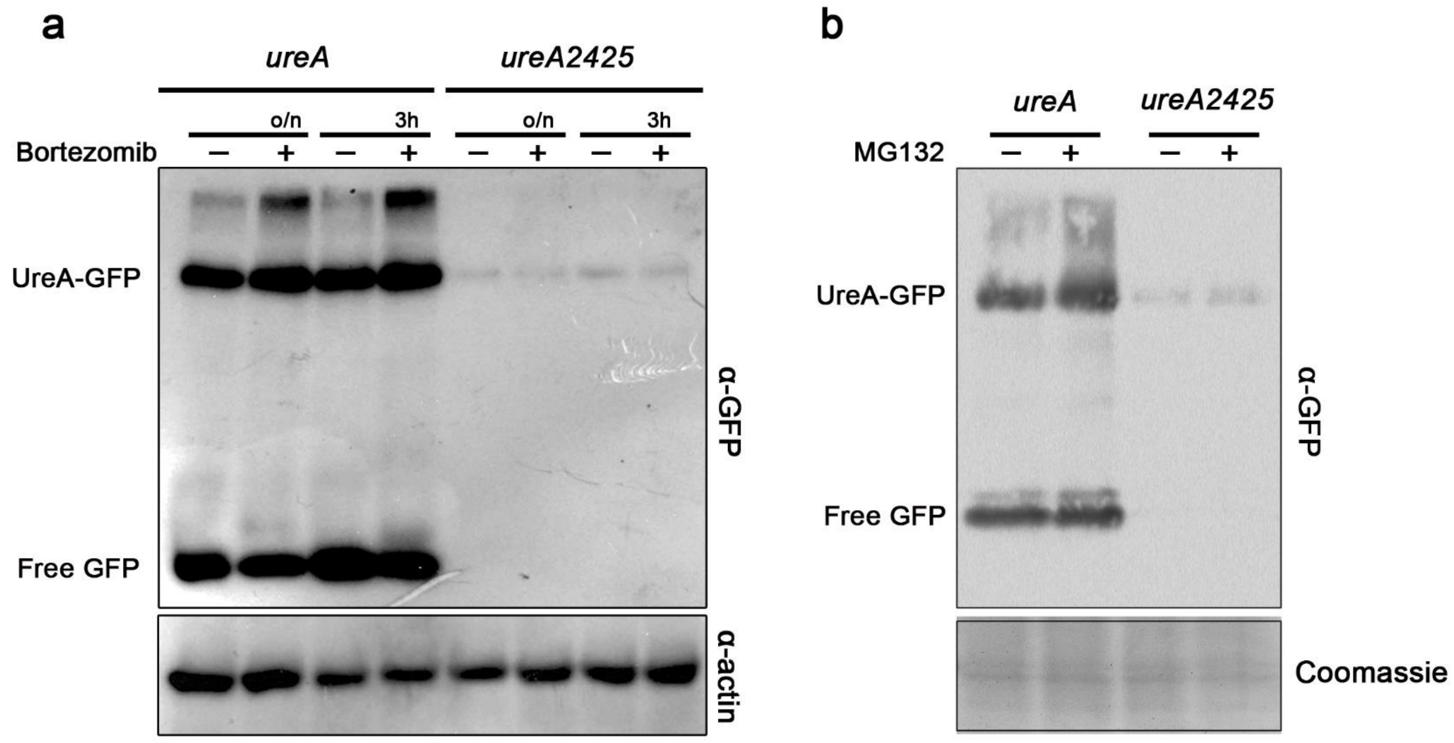

Supplementary Figure S4. Proteasome inhibition assays. Western blot analysis on total protein extracts of UreA-GFP wild type (*ureA*) and UreA2425-GFP (*ureA2425*) strains, in the presence (+) or absence (-) of proteasome inhibitors bortezomib (5 µM) **(a)** or MG132 (120 µM) **(b)**. In (b), results from overnight (o/n) or 3-hours (3 h) incubations with bortezomib are shown. See Materials and Methods for details. α-actin: anti-actin antibody; Coomassie: Coomassie staining. Detection of actin and Coomassie staining are shown as loading controls.

Supplementary Table S1. Relative synonymous codon usage (RSCU) (Sharp et al. 1986) calculated for the 160 highly expressed genes (data taken from Iriarte et al. 2012) and for all CDS in the genome of *Aspergillus nidulans*, *Aspergillus clavatus*, *Aspergillus flavus*, *Aspergillus fumigatus*, *Neosartorya fischeri*, *Aspergillus niger*, *Aspergillus oryzae* and *Aspergillus terreus*. All codon usage statistics were calculated with CodonW (Peden J. 2005, available at codonw.sourceforge.net/). The synonymous codon families studied in the present work are indicated in grey boxes. Synonymous mutations are indicated with green arrows.

RSCU calculated for 160 highly expressed genes.																	
		Aspergillus nidulans		*Aspergillus clavatus*		*Aspergillus flavus*		*Aspergillus fumigatus*		*Neosartorya fischeri*		*Aspergillus niger*		*Aspergillus oryzae*		*Aspergillus terreus*	
		Count	*RSCU*	*Count*	*RSCU*	*Count*	*RSCU*	*Count*	*RSCU*	*Count*	*RSCU*	*Count*	*RSCU*	*Count*	*RSCU*	*Count*	*RSCU*
**Phe**	UUU	593	0,43	429	0,31	501	0,36	596	0,42	486	0,35	596	0,37	505	0,37	391	0,28
	UUC	2163	1,57	2378	1,69	2291	1,64	2275	1,59	2326	1,65	2656	1,63	2260	1,64	2435	1,72
**Leu**	UUA	95	0,10	62	0,06	85	0,09	88	0,09	62	0,06	152	0,14	86	0,09	33	0,03
	UUG	758	0,79	674	0,68	940	0,96	829	0,82	774	0,78	867	0,78	940	0,96	504	0,51
	CUU	1396	1,45	1035	1,05	1348	1,38	1196	1,19	1120	1,14	1229	1,11	1334	1,37	806	0,81
	CUC	2037	2,11	2060	2,09	1828	1,87	1933	1,92	1953	1,98	2164	1,95	1820	1,87	2237	2,25
	CUA	223	0,23	103	0,10	164	0,17	194	0,19	149	0,15	191	0,17	170	0,17	79	0,08
	CUG	1287	1,33	1993	2,02	1515	1,55	1806	1,79	1862	1,89	2056	1,85	1505	1,54	2308	2,32
**Ile**	AUU	1418	1,11	1098	0,85	1359	1,07	1431	1,09	1319	1,01	1236	0,90	1353	1,07	978	0,79
	AUC	2305	1,81	2725	2,12	2371	1,87	2431	1,84	2512	1,93	2730	1,98	2351	1,86	2719	2,19
	AUA	94	0,07	42	0,03	83	0,07	95	0,07	70	0,05	176	0,13	79	0,06	37	0,03
**Met**	AUG	1394	1,00	1500	1,00	1437	1,00	1491	1,00	1491	1,00	1580	1,00	1433	1,00	1524	1,00
**Val**	GUU	1643	1,37	1343	1,08	1606	1,29	1527	1,22	1492	1,21	1490	1,12	1593	1,30	951	0,76
	GUC	2273	1,89	2635	2,11	2301	1,84	2457	1,97	2504	2,03	2754	2,07	2230	1,82	2820	2,25
	GUA	159	0,13	129	0,10	207	0,17	187	0,15	162	0,13	164	0,12	204	0,17	87	0,07
	GUG	731	0,61	888	0,71	877	0,70	822	0,66	789	0,64	926	0,69	865	0,71	1157	0,92
**Ser**	UCU	1078	1,38	887	1,12	1158	1,43	1088	1,33	1016	1,28	1205	1,27	1113	1,41	782	0,99
	UCC	1439	1,84	1585	2,01	1522	1,88	1441	1,76	1457	1,84	2049	2,16	1498	1,90	1820	2,30
	UCA	368	0,47	164	0,21	262	0,32	324	0,40	266	0,34	302	0,32	254	0,32	82	0,10
	UCG	535	0,68	815	1,03	581	0,72	724	0,89	721	0,91	659	0,70	551	0,70	811	1,03
**Pro**	CCU	1253	1,47	1064	1,23	1228	1,43	1229	1,39	1199	1,39	1143	1,15	1199	1,42	819	0,92
	CCC	1468	1,73	1847	2,14	1526	1,78	1641	1,86	1680	1,94	2080	2,09	1508	1,79	2059	2,32
	CCA	295	0,35	224	0,26	359	0,42	325	0,37	272	0,31	303	0,30	361	0,43	122	0,14
	CCG	387	0,46	313	0,36	312	0,36	331	0,38	312	0,36	461	0,46	307	0,36	545	0,62
**Thr**	ACU	1198	1,17	977	0,96	1307	1,29	1155	1,14	1117	1,12	1175	1,06	1268	1,27	863	0,85
	ACC	1979	1,93	2321	2,29	2061	2,03	2039	2,01	2069	2,08	2501	2,25	2048	2,04	2509	2,46
	ACA	495	0,48	368	0,36	360	0,36	450	0,44	407	0,41	296	0,27	352	0,35	126	0,12
	ACG	426	0,42	395	0,39	334	0,33	424	0,42	394	0,40	480	0,43	340	0,34	584	0,57
**Ala**	GCU	2367	1,50	2206	1,34	2431	1,49	2434	1,47	2412	1,45	2502	1,45	2410	1,50	1766	1,06
	GCC	2650	1,68	3197	1,95	2840	1,74	2890	1,75	2990	1,80	3231	1,88	2791	1,74	3782	2,27
	GCA	550	0,35	407	0,25	641	0,39	583	0,35	557	0,34	501	0,29	627	0,39	249	0,15
	GCG	745	0,47	760	0,46	616	0,38	703	0,43	678	0,41	658	0,38	601	0,37	855	0,51

**Tyr**	UAU	484	0,45	564	0,53	592	0,55	546	0,50	495	0,46	499	0,42	583	0,55	383	0,35
	UAC	1651	1,55	1578	1,47	1574	1,45	1646	1,50	1660	1,54	1881	1,58	1543	1,45	1784	1,65
**TER**	UAA	91	1,71	95	1,78	106	1,99	90	1,69	95	1,78	171	1,27	103	1,93	107	2,01
	UAG	46	0,86	47	0,88	37	0,69	46	0,86	46	0,86	92	0,69	34	0,64	34	0,64
**His**	CAU	320	0,46	336	0,47	400	0,56	379	0,51	317	0,44	354	0,43	384	0,55	285	0,40
	CAC	1071	1,54	1088	1,53	1023	1,44	1107	1,49	1123	1,56	1279	1,57	1023	1,45	1155	1,60
**Gln**	CAA	585	0,47	439	0,34	613	0,47	597	0,46	530	0,41	490	0,36	597	0,47	343	0,26
	CAG	1909	1,53	2126	1,66	2004	1,53	2020	1,54	2048	1,59	2261	1,64	1957	1,53	2290	1,74
**Asn**	AAU	479	0,37	492	0,37	542	0,40	612	0,45	536	0,40	468	0,33	534	0,40	294	0,22
	AAC	2140	1,63	2205	1,64	2158	1,60	2102	1,55	2143	1,60	2404	1,67	2132	1,60	2400	1,78
**Lys**	AAA	664	0,30	409	0,18	461	0,21	552	0,24	474	0,21	460	0,20	466	0,21	283	0,13
	AAG	3708	1,70	4066	1,82	4030	1,80	4008	1,76	4053	1,79	4142	1,80	3993	1,79	4158	1,87
**Asp**	GAU	1518	0,77	1756	0,88	1739	0,88	1668	0,84	1582	0,80	1545	0,75	1718	0,88	1394	0,70
	GAC	2407	1,23	2256	1,13	2222	1,12	2292	1,16	2351	1,20	2555	1,25	2179	1,12	2598	1,30
**Glu**	GAA	1437	0,61	1093	0,46	1393	0,57	1394	0,58	1329	0,55	1095	0,44	1376	0,57	1007	0,42
	GAG	3317	1,40	3707	1,55	3521	1,43	3431	1,42	3519	1,45	3908	1,56	3463	1,43	3799	1,58
**Cys**	UGU	193	0,55	182	0,50	240	0,68	207	0,55	171	0,47	306	0,66	249	0,70	193	0,52
	UGC	506	1,45	542	1,50	468	1,32	547	1,45	556	1,53	616	1,34	464	1,30	545	1,48
**TER**	UGA	23	0,43	18	0,34	17	0,32	24	0,45	19	0,36	140	1,04	23	0,43	19	0,36
**Trp**	UGG	820	1,00	842	1,00	823	1,00	850	1,00	837	1,00	930	1,00	822	1,00	844	1,00
**Arg**	CGU	1226	2,02	1250	2,03	1489	2,43	1343	2,12	1338	2,14	1486	2,15	1447	2,43	1323	2,09
	CGC	1378	2,27	1372	2,23	1168	1,91	1233	1,94	1240	1,98	1510	2,19	1136	1,91	1562	2,47
	CGA	316	0,52	168	0,27	178	0,29	265	0,42	227	0,36	177	0,26	174	0,29	135	0,21
	CGG	290	0,48	396	0,64	325	0,53	387	0,61	389	0,62	361	0,52	324	0,54	459	0,73
**Ser**	AGU	317	0,41	267	0,34	356	0,44	337	0,41	322	0,41	392	0,41	352	0,45	255	0,32
	AGC	955	1,22	1026	1,30	992	1,22	989	1,21	976	1,23	1075	1,14	973	1,23	994	1,26
**Arg**	AGA	215	0,35	403	0,65	293	0,48	404	0,64	384	0,61	400	0,58	279	0,47	243	0,38
	AGG	215	0,35	109	0,18	223	0,36	178	0,28	172	0,28	211	0,31	215	0,36	70	0,11
**Gly**	GGU	2444	1,83	2419	1,76	2618	1,91	2437	1,79	2491	1,83	2834	1,97	2599	1,91	2414	1,77
	GGC	1880	1,41	2181	1,59	1841	1,35	1982	1,45	1974	1,45	1897	1,32	1825	1,34	2256	1,66
	GGA	776	0,58	699	0,51	840	0,61	797	0,59	785	0,58	789	0,55	829	0,61	630	0,46
	GGG	237	0,18	188	0,14	176	0,13	238	0,18	197	0,15	242	0,17	179	0,13	151	0,11

RSCU calculated for all the annotated coding sequences.																	
		Aspergillus nidulans		*Aspergillus clavatus*		*Aspergillus flavus*		*Aspergillus fumigatus*		*Neosartorya fischeri*		*Aspergillus niger*		*Aspergillus oryzae*		*Aspergillus terreus*	
		Count	*RSCU*	*Count*	*RSCU*	*Count*	*RSCU*	*Count*	*RSCU*	*Count*	*RSCU*	*Count*	*RSCU*	*Count*	*RSCU*	*Count*	*RSCU*
**Phe**	UUU	73372	0,77	53906	0,65	83254	0,75	65058	0,73	67577	0,72	74306	0,72	79031	0,76	53176	0,64
	UUC	117834	1,23	112217	1,35	138357	1,25	112580	1,27	121345	1,29	131937	1,28	129459	1,24	112389	1,36
**Leu**	UUA	29141	0,37	17437	0,26	38831	0,44	22403	0,31	22704	0,30	29544	0,34	36915	0,44	14636	0,22
	UUG	69960	0,89	65756	0,96	95823	1,09	74222	1,01	77978	1,01	93213	1,07	91166	1,10	60106	0,89
	CUU	92982	1,18	64291	0,94	101643	1,15	77254	1,06	79142	1,03	89422	1,03	95940	1,15	56284	0,84
	CUC	122200	1,55	111243	1,62	121454	1,38	110312	1,51	118007	1,53	128157	1,48	113335	1,36	116174	1,73
	CUA	49144	0,62	29775	0,44	57096	0,65	36765	0,50	38290	0,50	51873	0,60	54018	0,65	27945	0,42
	CUG	108805	1,38	122419	1,79	114662	1,30	118130	1,61	126155	1,64	128424	1,48	107879	1,30	129053	1,92
**Ile**	AUU	91630	1,07	67041	0,92	106623	1,07	80558	1,02	83595	1,01	90383	1,00	100198	1,08	61427	0,87
	AUC	128099	1,49	131281	1,80	148606	1,50	127413	1,62	136084	1,64	141080	1,57	138491	1,49	132194	1,86
	AUA	38416	0,45	20152	0,28	42570	0,43	28285	0,36	28745	0,35	38625	0,43	40956	0,44	19315	0,27
**Met**	AUG	106205	1,00	96439	1,00	126037	1,00	101214	1,00	108342	1,00	116988	1,00	118373	1,00	96901	1,00
**Val**	GUU	86292	1,10	59079	0,85	97600	1,07	69435	0,94	72807	0,92	79639	0,94	91762	1,07	50917	0,73
	GUC	114661	1,46	110410	1,59	121889	1,34	113360	1,53	122842	1,55	118057	1,39	113390	1,33	115699	1,65
	GUA	35832	0,46	23373	0,34	44863	0,49	30422	0,41	31059	0,39	39363	0,46	43228	0,51	20438	0,29
	GUG	78328	0,99	85832	1,23	99052	1,09	83824	1,13	89537	1,13	102817	1,21	93403	1,09	93234	1,33
**Ser**	UCU	78224	1,09	57647	0,92	86984	1,10	68100	1,01	69536	0,99	81764	1,01	81562	1,10	52720	0,88
	UCC	83295	1,16	84865	1,36	96695	1,22	84332	1,25	89011	1,27	108276	1,34	90185	1,22	89725	1,49
	UCA	64002	0,89	42787	0,68	67299	0,85	53595	0,80	53778	0,77	65229	0,81	63457	0,86	33294	0,55
	UCG	72069	1,00	75862	1,21	77557	0,98	73980	1,10	78552	1,12	83166	1,03	71949	0,97	76845	1,28
**Pro**	CCU	82064	1,06	66220	0,96	92290	1,09	77998	1,08	81011	1,07	87258	0,99	86021	1,09	57987	0,85
	CCC	76403	0,99	85088	1,24	88472	1,05	79565	1,10	84007	1,11	101751	1,15	82172	1,05	88039	1,28
	CCA	72483	0,94	55102	0,80	83461	0,99	63095	0,87	64371	0,85	79122	0,89	77685	0,99	47007	0,69
	CCG	77689	1,01	68958	1,00	74048	0,88	68973	0,95	73856	0,97	85853	0,97	68748	0,87	81523	1,19
**Thr**	ACU	69798	0,91	52384	0,80	82990	0,96	63610	0,90	65616	0,88	72911	0,87	77689	0,97	45799	0,70
	ACC	91478	1,19	96332	1,46	110541	1,28	93099	1,32	99424	1,34	115924	1,38	102361	1,27	101658	1,56
	ACA	75154	0,98	52865	0,80	81636	0,95	62729	0,89	65227	0,88	71554	0,85	76309	0,95	41888	0,64
	ACG	70672	0,92	61925	0,94	70486	0,82	62797	0,89	67491	0,91	74745	0,89	65803	0,82	71901	1,10
**Ala**	GCU	113983	1,04	90712	0,92	130833	1,09	104928	1,02	110629	1,00	114884	1,01	122537	1,10	76803	0,79
	GCC	130207	1,18	134594	1,37	145819	1,22	129829	1,26	139151	1,26	146050	1,28	135033	1,21	145746	1,50
	GCA	96885	0,88	72115	0,73	107965	0,90	84720	0,82	90159	0,82	97773	0,86	101108	0,91	62166	0,64
	GCG	99016	0,90	96823	0,98	94011	0,79	93127	0,90	100688	0,91	97190	0,85	87745	0,79	103966	1,07
**Tyr**	UAU	62148	0,83	51367	0,83	80040	0,93	55369	0,83	58929	0,82	68586	0,86	74889	0,94	47288	0,74
	UAC	87199	1,17	72480	1,17	92045	1,07	78098	1,17	85168	1,18	90674	1,14	84744	1,06	80104	1,26
**TER**	UAA	3248	0,85	2361	0,78	3926	0,94	2508	0,76	2642	0,76	7903	0,66	3929	0,98	2150	0,73
	UAG	3823	1,00	2750	0,91	3639	0,87	3012	0,91	3320	0,95	7503	0,63	3541	0,88	2928	0,99

**His**	CAU	60230	0,99	52444	0,96	74387	1,06	58671	1,00	60713	1,00	72492	1,00	69438	1,06	49704	0,90
	CAC	61947	1,01	56489	1,04	66280	0,94	58147	1,00	61274	1,01	72859	1,00	61479	0,94	60209	1,10
**Gln**	CAA	81326	0,78	64667	0,70	99880	0,86	73499	0,75	76297	0,74	92445	0,84	92400	0,86	57360	0,65
	CAG	126077	1,22	119816	1,30	132398	1,14	121542	1,25	130546	1,26	128780	1,16	123503	1,14	119161	1,35
**Asn**	AAU	80416	0,85	64600	0,81	97924	0,90	74086	0,86	77685	0,85	79159	0,84	91125	0,90	53825	0,69
	AAC	108875	1,15	94093	1,19	119495	1,10	97690	1,14	104719	1,15	109924	1,16	110708	1,10	101271	1,31
**Lys**	AAA	86263	0,73	65200	0,63	98503	0,73	75508	0,68	78617	0,67	75697	0,66	91553	0,73	60181	0,61
	AAG	148806	1,27	142909	1,37	171243	1,27	145877	1,32	156646	1,33	153913	1,34	158973	1,27	136233	1,39
**Asp**	GAU	138140	0,97	125920	0,99	172282	1,06	129012	0,97	137633	0,97	146695	1,01	159928	1,06	109754	0,86
	GAC	148018	1,04	128171	1,01	153818	0,94	136403	1,03	147535	1,04	143477	0,99	141610	0,94	145043	1,14
**Glu**	GAA	133416	0,84	107616	0,77	157737	0,89	120045	0,82	127421	0,81	134156	0,85	146068	0,89	100756	0,75
	GAG	184868	1,16	173382	1,23	196797	1,11	173816	1,18	187838	1,19	180057	1,15	182276	1,11	166991	1,25
**Cys**	UGU	24332	0,75	20144	0,75	33649	0,91	24135	0,78	23989	0,76	37726	0,83	32421	0,92	22185	0,78
	UGC	40539	1,25	33447	1,25	40740	1,10	38011	1,22	39461	1,24	53541	1,17	38440	1,09	34907	1,22
**TER**	UGA	4367	1,15	4003	1,32	4922	1,18	4367	1,33	4507	1,29	20368	1,71	4593	1,14	3819	1,29
**Trp**	UGG	75567	1,00	63617	1,00	88031	1,00	69741	1,00	74622	1,00	91729	1,00	82965	1,00	65967	1,00
**Arg**	CGU	48763	0,91	42452	0,90	61576	1,08	48091	0,94	49043	0,93	57803	0,92	57893	1,08	42349	0,91
	CGC	83276	1,55	79656	1,69	78293	1,37	75638	1,49	79669	1,51	93135	1,48	72840	1,36	87895	1,89
	CGA	52492	0,98	44735	0,95	58559	1,03	50322	0,99	51732	0,98	62247	0,99	54891	1,02	41799	0,90
	CGG	56827	1,06	56847	1,21	59971	1,05	57680	1,13	60300	1,14	68579	1,09	56567	1,05	59424	1,28
**Ser**	AGU	50737	0,71	41196	0,66	62310	0,79	47682	0,71	49245	0,70	58315	0,72	58579	0,79	37642	0,63
	AGC	83271	1,16	73384	1,17	83927	1,06	75653	1,13	80442	1,15	86918	1,08	77619	1,05	71193	1,18
**Arg**	AGA	39447	0,73	32950	0,70	44203	0,78	39288	0,77	40493	0,77	48565	0,77	41986	0,78	26131	0,56
	AGG	41730	0,78	26101	0,55	39388	0,69	34545	0,68	35641	0,68	47223	0,75	37831	0,71	21742	0,47
**Gly**	GGU	82029	0,94	71301	0,94	108498	1,09	77642	0,97	83156	0,97	91467	0,97	102099	1,10	65255	0,86
	GGC	121893	1,40	113512	1,50	124618	1,26	112882	1,41	120176	1,40	122301	1,30	116124	1,25	119886	1,58
	GGA	77526	0,89	63465	0,84	95451	0,96	72086	0,90	77330	0,90	91186	0,97	89324	0,96	62568	0,82
	GGG	67590	0,78	54476	0,72	68464	0,69	58311	0,73	61699	0,72	71202	0,76	64463	0,69	56192	0,74

Supplementary Table S2. ***Aspergillus nidulans*** **strains used and constructed during this study**

Strain	*Genotype*
MVD 10A	*ureA::gfp::AFpyrG riboB2 pyrG89 pyroA4 ∆nkuA::argB veA1*
MVD 15	*yA2 ureA::gfp::AFpyrG riboB2 pyrG89 pyroA4 ∆nkuA::argB veA1*
MVD 13A	*ureA∆::riboB riboB2 pyrG89 pyroA4 ∆nkuA::argB veA1*
MVD 14A	*yA2 ureA∆::riboB riboB2 pyrG89 pyroA4 ∆nkuA::argB veA1*
MVD 2425*	*yA2 ureA2425 riboB2 pyrG89 pyroA4 ∆nkuA::argB veA1*
MVD 2425B*	*ureA2425 riboB2 pyrG89 pyroA4 ∆nkuA::argB veA1*
MVD 2425A*	*ureA2425A riboB2 pyrG89 pyroA4 ∆nkuA::argB veA1*
MVD 2425C*	*ureA2425C riboB2 pyrG89 pyroA4 ∆nkuA::argB veA1*
MVD 2424*	*ureA24 riboB2 pyrG89 pyroA4 ∆nkuA::argB veA1*
MVD 2525*	*ureA25 riboB2 pyrG89 pyroA4 ∆nkuA::argB veA1*

* This study.

Supplementary Table S3. Oligonucleotides used in this study

Primer Name	*5´to 3´Sequence*
Ure5-F	GAAACCTGGAGCAGTCGAAG
Ure3-R	CCCGATTTCTGAGACAAGGA
Ure5-N	GCACCGATGACAAGGGAGAT
Ure3-N	ACCAATGGATCTGGCACTAAAC
UreA2425-F	ACTGCTCCCCTCACCCA**G**GG**T**TTTGGGTACGGGATCA
UreA2425-R	TGATCCCGTACCCAAA**A**CC**C**TGGGTGAGGGGAGCAGT
UreA2425A-F	ACTGCTCCCCTCACCCA**G**GG**A**TTTGGGTACGGGATCA
UreA2425A-R	TGATCCCGTACCCAAA**T**CC**C**TGGGTGAGGGGAGCAGT
UreA2425C-F	ACTGCTCCCCTCACCCA**G**GG**C**TTTGGGTACGGGATCA
UreA2425C-R	TGATCCCGTACCCAAA**G**CC**C**TGGGTGAGGGGAGCAGT
UreA24-F	ACTGCTCCCCTCACCCA**G**GGGTTTGGGTACGGGATCA
UreA24-R	TGATCCCGTACCCAAACCC**C**TGGGTGAGGGGAGCAGT
UreA25-F	ACTGCTCCCCTCACCCAAGG**T**TTTGGGTACGGGATCA
UreA25-R	TGATCCCGTACCCAAA**A**CCTTGGGTGAGGGGAGCAGT
ureA_qPCR_3-F*	GGACAACGGCTACTACAACA
ureA_qPCR_3-R*	GAATAGCGAACCAGCAGAGA
actA-F*	GATCTGTACGGCAACATCGT
actA-R*	CGGTGATTTCCTTCTGCATAC

Mutated codons are underlined. Modified bases are highlighted in black.

* Primers used for qRT-PCR.
